# Liquiritin restores metabolic homeostasis in NAFLD by modulating AKT1/FOXO1 signaling

**DOI:** 10.3389/fphar.2026.1809617

**Published:** 2026-06-17

**Authors:** Zhirun Zhang, Sue Jiao, Tao Jiang, Ru Zhang, Zhuangzhuang Sun, Zhaopeng Zhang, Yanfang Pan, Junpeng Guo

**Affiliations:** 1 Changchun University of Chinese Medicine, Changchun, China; 2 Department of Basic Medicine, Shanxi University of Chinese Medicine, Xianyang, China; 3 Clinical College of Medicine, Changchun University of Chinese Medicine, Changchun, China

**Keywords:** AKT/FOXO1, immunity, lipid metabolism, liquiritin, NAFLD

## Abstract

**Background:**

Non-alcoholic fatty liver disease (NAFLD) is a prevalent chronic liver condition with a complex and multifactorial pathogenesis.

**Purpose:**

This study aimed to investigate the mecha-nism of liquiritin (LQ) in NAFLD for its treatment.

**Methods:**

This study integrates bioinformatics analysis with *in vitro* validation. Bulk RNA-seq analysis and single-cell dataset analysis were performed using Gene Expression Omnibus database resources. Molecular docking and molecular dynamics simulations were employed to evaluate the binding affinity of liquiritin with lipid metabolism-related proteins AKT and FOXO1. *In vitro* validation was conducted using the HepG2, AML12 and RAW 264.7 to investigate the mechanism by which liquiritin regulates lipid metabolic homeostasis in HepG2 cells and AML12 cells via the AKT/FOXO1 pathway through protein immunoblotting and immunofluorescence. The effects of liquiritin on mitochondrial autophagy in RAW 264.7 cells were also examined.

**Results:**

Bulk RNA-seq analysis identified FOXO1 as a key target, while single-cell analysis predicted interactions between hepatocytes and macrophages in non-alcoholic fatty liver disease (NAFLD). Molecular docking predicted a potential stable interaction between liquiritin and FOXO1. In HepG2 cells and AML12 cells, liquiritin improved NAFLD lipid metabolism disorders by regulating the AKT/FOXO1 pathway. Liquiritin also modulates the expression of mitophagy-associated proteins in RAW264.7 cells.

**Conclusion:**

Liquiritin improves lipid metabolism disorders in NAFLD by regulating the AKT/FOXO1 pathway, while also potentially influencing macrophage function. This suggests its potential as a candidate therapeutic agent for non-alcoholic fatty liver disease.

## 1 Introduction

The global prevalence of non-alcoholic fatty liver disease (NAFLD) among adults has approached 30% (Geary, 2025). Its development is intrinsically linked to metabolic syndrome and insulin resistance, involving complex metabolic abnormalities across multiple organs including the liver, adipose tissue, and gut (Zhou et al., 2025). The term non-alcoholic fatty liver disease (NAFLD) was introduced in 1986 but has been criticized for its exclusionary and potentially stigmatizing nature. In 2020, an international panel proposed replacing it with metabolic dysfunction-associated fatty liver disease (MAFLD) based on positive metabolic criteria, followed in 2023 by a multi-society Delphi consensus that established metabolic dysfunction-associated steatotic liver disease (MASLD) under a revised steatotic liver disease (SLD) classification (Nakano et al., 2024; Portincasa and Baffy, 2024). Importantly, substantial overlap exists between patients diagnosed under these nomenclatures, supporting the use of historical data.The core pathological driver of NAFLD is a comprehensive disruption of hepatic lipid metabolism (Arab et al., 2018; Watt et al., 2019; Nevola et al., 2023). Research indicates that under insulin resistance, sustained activation of the transcription factor SREBP-1c in the liver significantly enhances *de novo* lipid synthesis. This propels simple hepatic steatosis into the inflammatory state of non-alcoholic steatohepatitis (NASH) (Jiang et al., 2022; Zhao et al., 2022a; Yong et al., 2024).Current drugs primarily target metabolic and inflammatory pathways. However, the long-term effects of these drugs remain unclear. This has spurred research into multi-targeted, low-toxicity intervention strategies derived from natural products, necessitating future therapeutic development that simultaneously targets multiple key pathway nodes. Deepening our understanding of the multi-effect pharmacological mechanisms of natural products will provide crucial scientific foundations for developing novel, safe, and effective therapeutic agents.

Liquiritin (LQ), a major active component of the traditional herb licorice, has garnered considerable attention due to its lipid-lowering, anti-inflammatory, and antioxidant properties (Heidari et al., 2021; Liu et al., 2022; Guo et al., 2023). Liquiritin is a flavonoid O-glycoside derived from the roots of Glycyrrhiza (Chen et al., 2019). Accumulating evidence indicates that it exerts antioxidant and anti-inflammatory effects partly through the Nrf2/Keap1 and NF-κB/MAPK pathways (Gao et al., 2026). Moreover, total flavonoids from licorice, for which liquiritin is a major component, show hepatoprotective activity in liver injury models, and the aglycone form liquiritigenin has been reported to improve lipid accumulation and insulin resistance in NAFLD models via the PI3K/AKT pathway (Bao et al., 2023). Despite these promising properties, whether liquiritin itself directly regulates the AKT/FOXO1 axis—a key pathway in hepatic lipid metabolism—remains unexplored in NAFLD. Therefore, the present study was designed to investigate this mechanism.

The pathological process of NAFLD is closely associated with lipid metabolism disorders (Wang et al., 2022; Han et al., 2025). The AKT signaling axis acts as a key downstream effector of insulin, maintaining metabolic homeostasis by regulating the activity of the critical transcription factor FOXO1, which influences mitochondrial function and oxidative stress (Duan et al., 2021; Jaiswal et al., 2021; Chen et al., 2022). Dysfunction of the AKT/FOXO1 axis leads to abnormal activation and nuclear translocation of FOXO1. This drives excessive expression of lipogenic genes (e.g., SREBP-1c, ACC, FASN) while suppressing mitochondrial biogenesis and fatty acid oxidation, ultimately resulting in a vicious cycle of hepatic lipid accumulation, oxidative damage, and insulin resistance (Xu et al., 2021; Li et al., 2022; Xie et al., 2023). Therefore, targeting and modulating the functional balance of the AKT/FOXO1 axis may represent a novel strategy to improve lipid metabolism disorders in NAFLD.

This study employs a combined approach involving bioinformatics analysis and experimental validation to investigate the molecular mechanisms underlying NAFLD. Initially, we conducted a comprehensive bioinformatics analysis of the transcriptomic dataset GSE135251 from the Gene Expression Omnibus (GEO) database, with a focus on Kyoto Encyclopedia of Genes and Genomes (KEGG) and Gene Ontology (GO) enrichment analyses. This approach revealed significant alterations in the FOXO signaling pathway and lipid metabolism-related genes (Dong et al., 2022; Li et al., 2025a). Notably, the expression levels of AKT1, FOXO1, CPT1A, SREBF1, FASN, and ACACA were analyzed. This analysis revealed a marked downregulation of FOXO1 and CPT1A in NAFLD samples, while SREBF1, FASN, and ACACA were upregulated, indicating a disruption in lipid metabolism associated with the FOXO1 signaling pathway. Subsequently, transcription factor analysis was conducted, and a transcription factor expression network for the aforementioned genes was constructed, indicating that these genes exist within a highly interconnected and complex regulatory system. Furthermore, we performed cell communication analysis on single-cell datasets GSE174748, GSE189175, and GSE212837, which revealed strong interactions between hepatocytes and Kupffer cells, particularly within the DHEAS, Netrin, and PROS signaling networks. Relevant research indicates that there is a close relationship of the Netrin and PROS signaling networks with hepatic energy metabolism and liver injury (Huang et al., 2023; Wang et al., 2025), with the Netrin signaling network notably influencing macrophage autophagy (Zhao et al., 2022b; Ji et al., 2025; Kim et al., 2025). This multifaceted approach not only elucidates the complex interactions involved in NAFLD but also lays the groundwork for future research directions.

Subsequently, we used network pharmacology methods to predict potential protein targets of LQ related to NAFLD, identifying AKT1 as the primary candidate target. Subsequently, molecular docking and molecular dynamics simulations were used to assess the potential for direct binding between LQ and AKT1 and FOXO1 proteins. Results showed stable binding conformations between LQ and AKT1 and FOXO1 proteins. To validate these predictions, we conducted *in vitro* experiments using differentiated cell models. In a free fatty acid-induced HepG2 and AML12 hepatocyte steatosis model, LQ treatment significantly reduced lipid accumulation and cellular damage (Negi et al., 2021). Mechanistic investigations via Western blot (WB) and immunofluorescence (IF) revealed that LQ suppresses the expression of the key lipid synthesis protein SREBP-1c through the AKT1/FOXO1 signaling axis. This inhibition subsequently downregulated the expression of downstream lipid synthesis-related effector proteins ACC and FASN, while promoting the expression of CPT1A, a key protein in fatty acid β-oxidation (Wu et al., 2022; Li et al., 2025c). In a high-fat model of RAW264.7 cells, LQ influenced the expression of mitochondrial autophagy-related proteins P62 and LC3 (Wu et al., 2024). This suggests that under NAFLD conditions, alterations in hepatocytes may affect macrophages, potentially contributing to metabolic disorders and autophagy dysfunction. Thus, this study suggests that LQ may exerts therapeutic effects primarily through targeting the AKT1/FOXO1 signaling axis. This mechanism collectively contributes to alleviating hepatic steatosis by inhibiting excessive lipid synthesis, promoting fatty acid oxidation, and modulating macrophage function.

However, this study has several limitations. First, *in vivo* validation using animal models has not yet been performed. Second, the specific deacetylation sites of FOXO1 regulated by LQ warrant further detailed investigation. Additionally, the interactions within the immune microenvironment indicated by single-cell data analyses need to be validated in co-culture models. Future studies will establish NAFLD animal models to evaluate the long-term efficacy of LQ. They will also use gene editing techniques to validate its mechanism of action and the functional necessity of key targets, and explore the molecular mechanisms by which LQ regulates metabolic crosstalk between hepatocytes and macrophages. These findings not only provide a theoretical foundation for incorporating LQ into targeted therapeutic candidates for NAFLD but also pave the way for novel pathways in developing precision intervention strategies against metabolic liver diseases based on natural products.

## 2 Materials and Methods

### 2.1 Data and preprocessing

The NAFLD transcriptome dataset GSE135251 was obtained from the Gene Expression Omnibus (GEO) database at the National Center for Biotechnology Information (NCBI). This dataset, generated via RNA sequencing technology, comprises 216 samples: 10 CON samples and 206 NAFLD pathological samples. To assess overall data quality and potential batch effects, PCA plots were generated from raw expression profiles, while differential expression analysis was performed using the DESeq2 software package. Genes with *p*-values < 0.05 and |log_2_FC| > 1 were considered significantly differentially expressed. Based on these identified genes, KEGG pathway and GO biological process enrichment analyses were conducted to explore functional associations. Volcano plots were used to visualize the overall distribution of differentially expressed genes. Key lipid metabolism-related genes—AKT1, FOXO1, CPT1A, SREBF1, FASN, and ACACA—were highlighted to illustrate their differential expression in NAFLD. We integrated three liver single-cell RNA sequencing datasets (GSE174748, GSE189175, and GSE212837) in this study. Following batch effect correction and quality control, we performed cellular clustering analysis. Using hallmark genes established in authoritative databases such as CellMarker 2.0 and scLiverDB, cell clusters were annotated into major types including hepatocytes, Kupffer cells and cholangiocytes. Subsequently, cell cycle analysis was performed within each annotated cell subpopulation. To uncover disease-associated cellular state transitions, intercellular communication analysis was conducted on key populations like hepatic macrophages. Results revealed a high-intensity signaling interaction network between hepatocytes and Kupffer cells. Ligand-receptor analysis further elucidated the primary functional mechanism alterations between these two cell types.

### 2.2 Transcription factor analysis

To systematically identify transcription factors regulating key differentially expressed genes, this study integrated information on binding sites and regulatory elements from multiple authoritative databases, such as FIMO_JASPAR, GTRD, COr_GTEX, hTFtarget, ChIP_Atlas, and ENCODE. Through integrated analysis, potential transcription factors predicted to bind target gene promoter regions were identified. Subsequently, a transcription factor–target gene regulatory network was constructed, revealing the upstream regulatory mechanisms controlling the expression of lipid metabolism genes.

### 2.3 Network pharmacology analysis

Network pharmacology approach was used to identify potential targets and mechanisms of action for LQ in treating NAFLD. First, NAFLD-associated target genes were collected from disease databases (including DisGeNET, GeneCards, OMIM, and TTD), and potential targets for LQ were obtained from databases such as HERB, IDmapping, and SwissTargetPrediction. By identifying the intersection between disease targets and drug targets, a common target gene set was identified to enable prediction of the key signaling pathways and biological processes involved in LQ’s action against NAFLD. Based on significant transcriptomic differences and pharmacogenomic predictions, AKT/FOXO1 was identified as a target for further research.

### 2.4 Molecular docking and dynamics simulation analysis

The binding potential of LQ with the core target protein was validated through molecular docking and molecular dynamics simulations. The three-dimensional structure of the LQ molecule was obtained from the PubChem database: CAS No. 551-15-5. The SMILES notation of LQ is C1 [C@H](OC2 = C(C1 = O)C=CC(=C2)O)C3 = CC = C(C=C3)O [C@H]4 [C@@H]([C@H]([C@@H]([C@H](O4)CO)O)O)O. The crystal structures of the core target proteins AKT1 (PDB ID: 6CCY) and FOXO1 (PDB ID: 4LG0) were downloaded from the Protein Data Bank (PDB). The preprocessing steps involved removing water molecules and small-molecule ligands, adding hydrogen atoms, and assigning charges to the proteins. Molecular docking was performed using software such as AutoDock Vina to evaluate binding modes and affinities. The optimal docking conformation was selected for subsequent molecular dynamics simulations. Perform molecular dynamics simulations on LQ and the target protein under the previously defined experimental conditions (Jiang et al., 2025).

### 2.5 Drugs and reagents

LQ (purity ≥ 99.99%, HY-N0376) was obtained from MCE (Shanghai, China). Sodium Palmitate/Sodium Oleate Kit for Experimental Use (KT003) was purchased from Kunchuang Technology Development Co., Ltd. (Xi’an, China). Oil Red O staining (G1262) was purchased from Beyotime Beijing Solarbio Science & Technology Co.,Ltd. (Beijing, China). Antibodies for AKT (10176-2-AP), Phospho-AKT (66444-1-Ig), FOXO1 (18592-1-AP), Phospho-FOXO1 (28757-1-AP), CPT1 (15184-1-AP), P62 (18420-1-AP), LC3 (14600-1-AP), PINK1 (23274-1-AP), Parkin (14060-1-AP), ACC (21923-1-AP), and FASN (10624-2-AP) were acquired from Proteintech (Boston, MA, USA). Antibodies for SREBP-1c (AF6283) were purchased from Affinity Biosciences (Jiangsu, China).

### 2.6 Cell culture and treatment

The human hepatocellular carcinoma line HepG2 (SCSP-510) was maintained at 37 °C under 5% CO_2_ in Dulbecco’s modified Eagle’s medium (DMEM) supplemented with 10% fetal bovine serum and 1% penicillin–streptomycin (Cytiva, USA). The mouse hepatocyte cell line AML12 (SCSP-550) was maintained at 37 °C under 5% CO_2_ in Dulbecco’s Modified Eagle Medium/ Nutrient Mixture F-12 (DMEM/F-12) supplemented with 10% fetal bovine serum, 1% penicillin–streptomycin (Cytiva, USA) and 1% ITS Liquid Media Supplement (100×) (Sigma,USA).The murine macrophage cell line RAW 264.7 (SCSP-5036) was maintained at 37 °C under 5% CO_2_ in Dulbecco’s modified Eagle’s medium (DMEM) supplemented with 15% fetal bovine serum. The optimal working concentration of LQ for both HepG2, AML12 and RAW 264.7 cells was determined using the CCK-8 assay. All cell lines were obtained from the National Collection of Authenticated Cell Cultures. For experiments, HepG2 was allocated to five groups: (a) blank control; (b) FFA (free fatty acid) model; (c) LQ low (30 μM), (d)medium (60 μM), and (e) high (120 μM) dose. Cells were exposed to 500 μM oleic acid (OA) plus 250 μM palmitic acid (PA) for 24 h to establish the lipotoxic model, followed by 24 h treatment with LQ at the indicated concentrations. For the AKT inhibitor experiment, HepG2 was divided into four groups: (a) blank control; (b) FFA model; (c) FFA + LQ high dose (120 μM); (d) FFA + LQ high dose (120 μM) + MK-2206 (5 μM). For the FOXO1 inhibitor experiment, cells were divided into four groups: (a) blank control; (b) FFA model; (c) FFA + LQ high dose (120 μM); (d) FFA + LQ high dose (120 μM) + AS1842856 (1 μM). Cells in the inhibitor experiments were subjected to the same FFA induction (500 μM OA + 250 μM PA for 24 h) followed by 24 h co-treatment with LQ and the respective inhibitors. AML12 cells was allocated to five groups: (a) blank control; (b) FFA (free fatty acid) model; (c) LQ low (30 μM), (d) medium (60 μM), and (e) high (120 μM) dose. Cells were exposed to 1000 μM oleic acid (OA) plus 500 μM palmitic acid (PA) for 24 h to establish the lipotoxic model, followed by 24 h treatment with LQ at the indicated concentrations. RAW 264.7 cells were allocated to five groups: (a) blank control; (b) FFA (free fatty acid) model; (c) LQ low (20 μM), (d) medium (40 μM), and (e) high (80 μM) dose. Cells were exposed to 100 μM oleic acid (OA) plus 50 μM palmitic acid (PA) for 12 h to establish the lipotoxic model, followed by 12 h treatment with LQ at the indicated concentrations. All experiments had three biological replicates (n = 3).

### 2.7 Cell viability assay

An enhanced Cell Counting Kit-8 (CCK-8; NCM Biotech, Suzhou, China) was used to measure cell viability. HepG2 cells, AML12 cells and RAW 264.7 cells were seeded in 96-well plates at a density of 5000 cells per well and cultured with different treatments. The cells were then incubated with the CCK8 assay mixture for 2 h, and the absorbance was measured at 450 nm. For each condition, six independent biological duplicates were assessed. All experiments had three biological replicates (n = 3).

### 2.8 Lipid peroxidation assay

Lipid peroxidation was assessed with BODIPY 581/591 C11(Servicebio, China). HepG2 cells were seeded at 1 × 10^4^ cells per well in 24-well plates and subjected to the indicated treatments. After staining, cells were incubated with 5 µM C11-BODIPY for 20 min at 37 °C, washed twice with ice-cold PBS, and immediately imaged under a fluorescence microscope (Leica THUNDER Imaging Systems, Germany). All experiments had three biological replicates (n = 3).

### 2.9 Reactive oxygen species (ROS) staining

HepG2 cells was seeded in 24-well plates and cultured until 50%-70% confluence. After two washes with PBS, the cells were loaded with DCFH-DA working solution (Servicebio, China) and incubated for 30 min at 37 °C in the dark under 5% CO_2_. Following two additional PBS washes, the labeled cells were immediately observed under a fluorescence microscope (Leica THUNDER Imaging Systems, Germany). All experiments had three biological replicates (n = 3).

### 2.10 Detection of mitochondrial superoxide (MitoSOX red)

After routine culture or experimental treatment, cells were washed three times with PBS. Prewarmed MitoSOX Red working solution (Servicebio, China) was then added, and the cells were incubated at 37 °C for 20 min in the dark. The probe was removed, and the cells were washed 3 times with PBS. Fluorescence images were acquired using the DsRed channel of a fluorescence microscope (Leica THUNDER Imaging Systems, Germany). All experiments had three biological replicates (n = 3).

### 2.11 Oil red O (ORO) staining

HepG2 cells were seeded on glass slides placed in 6-well plates and cultured to 80% confluence. The cells were then exposed to 500 μM oleic acid (OA) plus 250 μM palmitic acid (PA) free fatty acids (sodium oleate: sodium palmitate = 2:1) with or without LQ (30, 60, and 120 μM). After 24 h, cells were washed twice with PBS and fixed with 4% paraformaldehyde. Subsequently, the cells were immersed in 60% isopropanol and stained with Oil Red O working solution. Stained cells were observed and photographed under a light microscope (ThermoFisher EVOS™ M5000 Imaging System, USA). All experiments had three biological replicates (n = 3).

### 2.12 Western blotting

Cells were homogenized in RIPA lysis buffer (Beyotime, China) containing protease and phosphatase inhibitors (Beyotime, China) for protein extraction. Protein concentrations were quantified using a BCA assay kit (Beyotime, China). After separation by sodium dodecyl sulfate-polyacrylamide gel electrophoresis (SDS-PAGE), proteins were transferred onto PVDF membranes (Millipore, Germany). The membranes were blocked with 5% non-fat milk for 1 h and then incubated overnight at 4 °C with the indicated primary antibodies ((Materials and Methods 2.5). Following three washes with TBST, the membranes were incubated with HRP-conjugated secondary antibodies. Immunoreactive bands were visualized using an enhanced chemiluminescence reagent kit on a chemiluminescence imaging system (Shenhua Technology, SH-Magic 523, Hangzhou, China). Band intensities were analyzed with ImageJ software, and β-actin served as the loading control.

### 2.13 Immunofluorescence staining

HepG2 and RAW264.7 cells were fixed with 4% paraformaldehyde and permeabilized with 0.1% Triton X-100 for 15 min. Incubate the cells with the primary antibody overnight at 4 °C. For antibody details, see Materials and Methods, Section 2.5. Immunofluorescence images were acquired with a laser confocal scanning microscope (Leica THUNDER Imaging Systems, Germany). Mean fluorescence intensity was quantified using ImageJ software.

### 2.14 Statistical analysis

All experiments had at least three independent biological replicates (n = 3). Data are presented as mean ± SD, analyzed with GraphPad Prism 10.0 (GraphPad Software, Inc.). One-way ANOVA followed by Tukey’s *post hoc* test was used for multiple comparisons. P < 0.05 was considered significant. Exact replicate numbers are given in the figure legends.

## 3 Results

### 3.1 Transcriptomic analysis of NAFLD reveals a lipid metabolic imbalance

**FIGURE 1:**
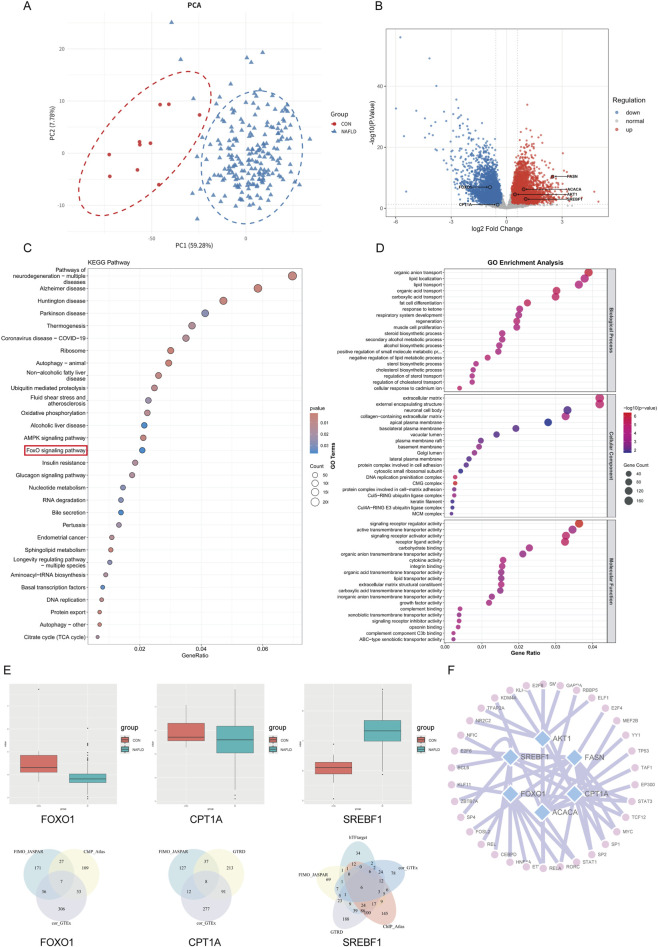
Bulk transcriptomic and transcription-factor analyses based on the GEO dataset. **(A)** PCA. **(B)** Volcano plot of differentially expressed genes. **(C)** KEGG enrichment analysis. **(D)** GO enrichment analysis. **(E)** Gene expression analysis and Analysis of transcription factors regulating key genes: *FOXO1*, *CPTA1*, and *SREBF1*. **(F)** Transcription factor co-expression network analysis.

First, data quality control was performed on the GSE135251 dataset. The first two principal components of principal component analysis (PCA) distinctly separated control (CON) samples from NAFLD samples, indicating significant transcriptomic differences between the two groups (Figure 1A). Following differential expression analysis with DEseq2, KEGG pathway enrichment analysis revealed a significant enrichment of differentially expressed genes in the FOXO signaling pathway (P < 0.05) (Figure 1C). GO biological process analysis identified enriched terms such as “lipid localization”, “lipid transport” and “negative regulation of lipid metabolic process” (Figure 1D) (Berardo et al., 2023), indicating heightened activity of these processes in NAFLD. The volcano plot illustrated the differential expression of key genes associated with lipid metabolism—AKT1, FOXO1, CPT1A, SREBF1, FASN, and ACACA—in NAFLD (Figure 1B). Analysis of differential gene expression levels further confirmed these changes: compared to the CON group, the expression of *FOXO1* and *CPT1A* was significantly reduced in the NAFLD group (Gao et al., 2022; Zhang et al., 2024b), indicating suppressed fatty acid β-oxidation in NAFLD. Conversely, *SREBF1* and its downstream target genes *FASN* and *ACACA* (Figure 1E) exhibited sustained, significant upregulation, indicating elevated expression of lipid synthesis-related genes in NAFLD (Parafati et al., 2024). These findings suggest that genes promoting fatty acid β-oxidation are suppressed in NAFLD, while pathways driving lipid synthesis are highly active. These findings suggest that FOXO1 likely plays a central regulatory role in this metabolic imbalance (Uehara et al., 2022). Given its potential key role in lipid metabolism, FOXO1 represents a highly promising therapeutic target for the treatment of NAFLD.

### 3.2 A transcription factor regulatory network in NAFLD

To elucidate the regulatory mechanisms of these key genes, we integrated transcription factor binding information from multiple authoritative databases, including JASPAR, hTFtarget, ChIP-Atlas, and ENCODE (Pratt et al., 2021; Ovek Baydar et al., 2025; Berrouayel and del Peso, 2026). The detailed information of transcription factor-target gene interactions, including binding site locations, detection methods, and supporting evidence, is summarized in Table X. We constructed a transcription factor-target gene regulatory network centered on *AKT1*, *FOXO1*, *CPT1A*, *SREBF1*, *ACACA*, and *FASN* (Figure 1F). Network analysis revealed these genes operate within a highly interconnected and complex regulatory system. The promoter region of AKT1 appears to be co-regulated by multiple universal transcription and signaling response factors, including *CTCF*, *SP1*, and *RELA* (Wan et al., 2021; Wang et al., 2021). *FOXO1* is predicted to be regulated by factors such as *KLF11*, *STAT3*, and *ETV6*, all of which are closely associated with inflammatory and metabolic stress responses (Ahmed et al., 2024; Albalawi and Khateeb, 2025; Li et al., 2025b). Importantly, the lipogenic genes *SREBF1* and *FASN* are not only transactivated by their own transcription factor *SREBF1* but may also be co-regulated by proto-oncogene/proliferation-associated factors such as *MYC* and *SP1*(Augustin et al., 2023). Furthermore, the association of *CPT1A* with factors like *TP53* and *MYC*(Nishida et al., 2023; Sabe, 2023; Wang et al., 2024b) suggests its expression may be regulated by cell cycle and stress states (Figure 1E). This network visually demonstrates that key genes driving NAFLD-associated lipid metabolism disorders undergo multi-layered, crosstalk regulatory control, with extensive involvement of numerous inflammation- and proliferation-related transcription factors. Notably, AKT1 and FOXO1 emerge as central nodes within this regulatory network, suggesting that they may serve as key hubs for therapeutic intervention. These findings provide crucial insights into the transcriptional regulatory basis of NAFLD and highlight potential molecular targets for drug development.

### 3.3 Single-cell transcriptomics reveals hepatocyte-kupffer cell crosstalk in NAFLD

**FIGURE 2:**
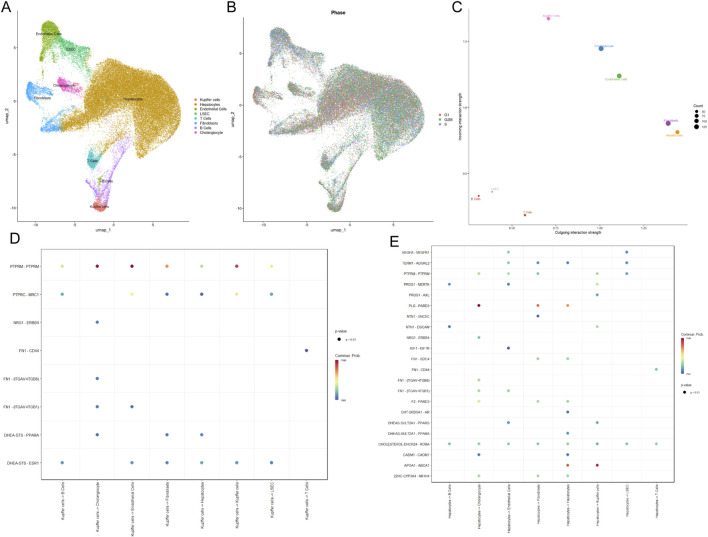
Single-cell annotation, cell cycle and communication analysis. **(A)** Single-cell clusters. **(B)** Cell cycle **(C)** Cell interaction intensity. **(D)** Kupffer cell ligand. **(E)** Hepatocytes ligand.

**FIGURE 3:**
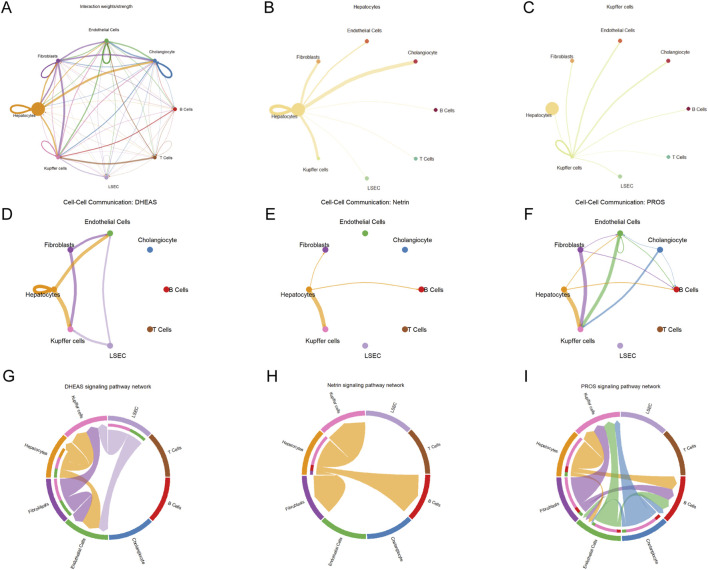
Single-Cell Communication and Pathway Analysis. **(A)** Interaction weights/strength. **(B)** Hepatocytes Communication. **(C)** Kupffer cell Communication. **(D)** Cell-Cell Communication: DHEAS. **(E)** Cell-Cell Communication: Netrin. **(F)** Cell-Cell Communication: PROS. **(G)** DHEAS Signaling Network Communication Network. **(H)** Netrin Signaling Network Communication Network. **(I)** PROS Signaling Network Communication Network.

To further investigate the mechanisms underlying non-alcoholic fatty liver disease (NAFLD), this study integrated three liver single-cell transcriptomic datasets (GSE174748, GSE189175, and GSE212837). Following quality control and data integration, eight cell subpopulations were jointly identified and annotated (Figure 2A) based on known marker genes (Supplementary Material Figure S3). Subsequently, cell cycle analysis was performed (Figure 2B). Cell communication analysis revealed strong signaling interactions between hepatocytes and Kupffer cells (Figures 2C, 3A). Receptor-targeted heatmap analysis (Supplementary Material Figure S4) was subsequently performed, followed by receptor analysis (Figures 2D,E) and communication analysis (Figures 3B,C) focusing on Kupffer cells and hepatocytes. This analysis highlighted the ligands DHEAS (Figure 3D), Netrin (Figure 3E), and PROS (Figure 3F). Corresponding communication analysis heatmaps—DHEAS signaling network (Supplementary Material Figure S5), Netrin signaling network (Supplementary Material Figure S6), and PROS signaling network (Supplementary Material Figure S7)—further confirmed robust interactions. To explore these pathways in depth, cell communication analysis based on genes and pathways was performed (Figures 3G–I). Results indicate that in NAFLD, the interactive signaling between hepatocytes and Kupffer cells exhibits high correlation. At the hepatocyte level, Netrin-1 promotes inflammation and fibrosis in the liver, potentially exacerbating hepatic metabolic inflammation and insulin resistance by recruiting macrophages (Kupffer cells) (Mentxaka et al., 2022). PROS1 exhibits anti-inflammatory and anti-apoptotic effects, regulating hepatic lipotoxicity and inflammation to drive NAFLD progression (Hörber et al., 2021; Iglesias Morcillo et al., 2023). At the Kupffer cell level, DHEAS and Netrin frequently correlate with autophagy (Lee et al., 2015; Vegliante et al., 2016; Bai et al., 2017; Chen et al., 2020; Gonulalan and Tanrıkulu, 2021; Tumilovich et al., 2024; Wang et al., 2024a). These analyses provide a comprehensive explanation for the strong interaction and crosstalk between hepatocytes and Kupffer cells in NAFLD, which collectively drive the pathological progression. Importantly, several of these identified signaling molecules (e.g., Netrin-1, PROS1) have been implicated in the regulation of AKT and FOXO1 pathways, suggesting that hepatocyte-Kupffer cell crosstalk may influence the AKT/FOXO1 signaling axis during NAFLD progression. This further supports the rationale for investigating therapeutic agents that modulate this key pathway.

### 3.4 Network pharmacology reveals the action targets of NAFLD and LQ

**FIGURE 4:**
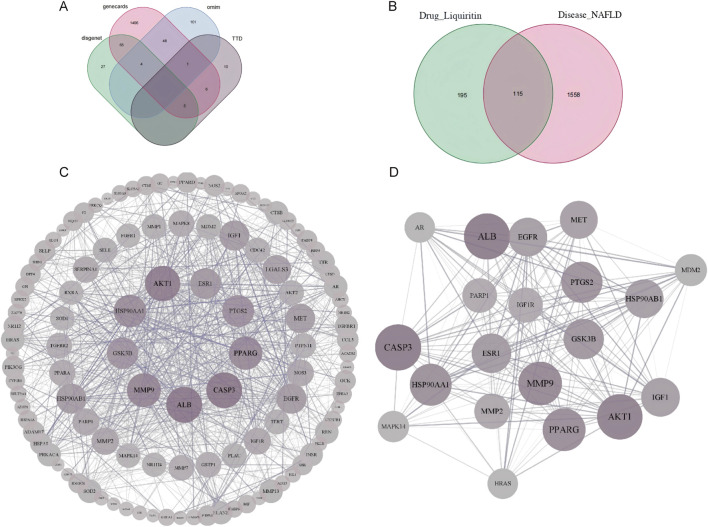
Network pharmacology analysis and molecular docking. **(A)** Venn diagram of NAFLD-related targets. **(B)** Venn diagram of drug-disease common targets. **(C)** PPI network of key proteins. **(D)** Top 20 hub proteins in the PPI network.

To identify potential targets for LQ in the treatment of non-alcoholic fatty liver disease (NAFLD), we conducted a network pharmacology analysis. LQ-associated targets were sourced from the TCMSP database and the SwissTargetPrediction platform; following screening based on probability scores, a total of 195 targets were identified. Targets associated with NAFLD were retrieved from the DisGeNET, GeneCards, OMIM and TTD databases, yielding a total of 1,558 targets (Figure 4A). The intersection of the two sets of targets revealed 115 common targets (Figure 4B). A protein-protein interaction network was constructed using the STRING database (confidence ≥ 0.7), and visualised using Cytoscape (Figure 4C). AKT was identified as the core target using the CytoHubba plugin in conjunction with the degree centrality algorithm (Figure 4D). The results indicate that AKT, as a hub target, is expected to play a central role in this network. Furthermore, an interaction exists between AKT1 and FOXO1; this finding is consistent with our transcriptomic and regulatory network analysis results. Taken together, this suggests that LQ may exert its therapeutic effects on non-alcoholic fatty liver disease (NAFLD) by modulating the AKT/FOXO1 signalling axis.

### 3.5 Molecular docking and dynamics simulations confirm stable binding of LQ to AKT1/FOXO1

**FIGURE 5:**
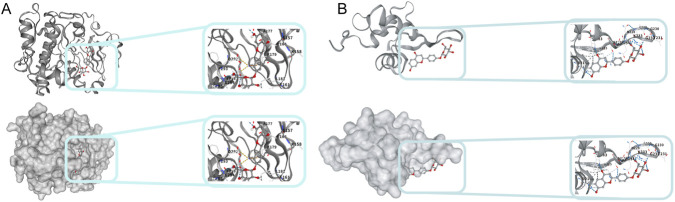
Molecular docking. **(A)** AKT1 Molecular Docking. **(B)** FOXO1 Molecular Docking.

**FIGURE 6:**
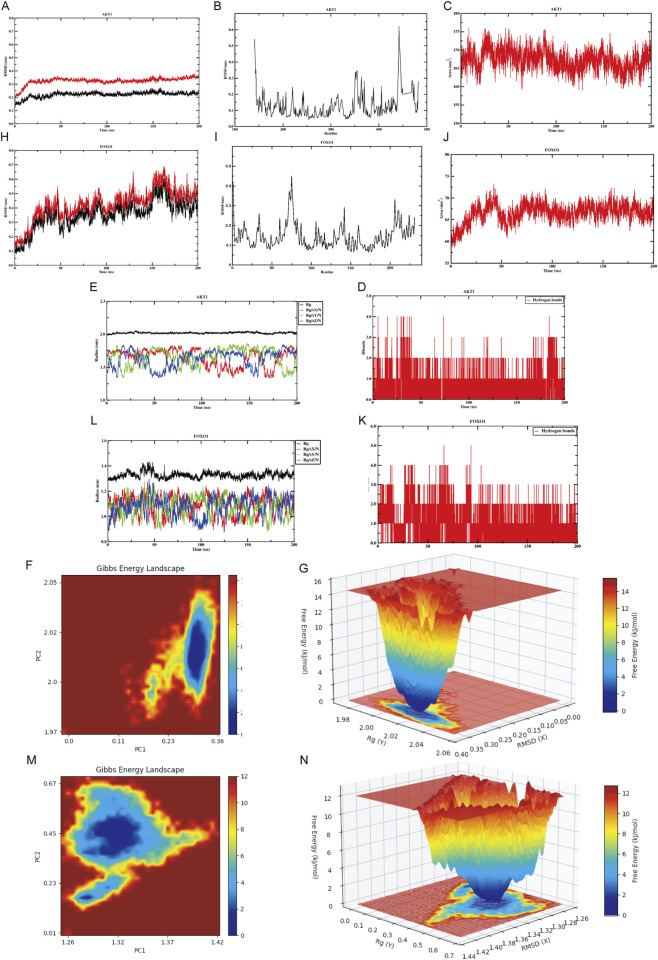
Molecular dynamics simulation of AKT1 and FOXO1. **(A)** RMSD of AKT1. **(B)** RMSF of AKT1. **(C)** SASA of AKT1. **(D)** Hbond of AKT1. **(E)** Rg of AKT1. **(F,G)** Free energy landscape plot of AKT1. **(H)** RMSD of FOXO1. **(I)** RMSF of FOXO1. **(J)** SASA of FOXO1. **(K)** Hbond of FOXO1. **(L)** Rg of FOXO1. **(M,N)** Free energy landscape plot of FOXO1.

Molecular docking simulations were performed to model the binding of LQ to the core target proteins AKT1 (Figure 5A) and FOXO1 (Figure 5B). Results indicate that LQ binds stably at or adjacent to the binding sites of both proteins. The calculated binding free energies are −8.2 kcal/mol for the AKT1 complex and −7.9 kcal/mol for the FOXO1 complex. Molecular dynamics simulations were conducted to assess the binding stability. The root means square deviation (RMSD) of the LQ-AKT1 complex stabilized at approximately 50 nanoseconds (Figure 6A), while the RMSD of the LQ-FOXO1 complex also stabilized at approximately 50 nanoseconds (Figure 6H), indicating structural stability of the complexes. Analysis of residue-average free energy RMSF (Figures 6B,I) and average surface area SASA (Figures 6C,J) provided insights into the binding process. Hydrogen bond energies (Figures 6D,K) and radius of gyration (Rg) measurements (Figures 6E,L) both supported the stability of the bound complexes. Furthermore, free energy landscape analysis (Figures 6F,G,M,N) detailed the associated energy changes, confirming the presence of persistent, stable interactions at the binding interface. These computational results suggest that LQ can potentially form stable interactions with AKT1 and FOXO1 proteins, providing a structural basis for further functional studies.

### 3.6 LQ alleviates hepatocyte injury

**FIGURE 7:**
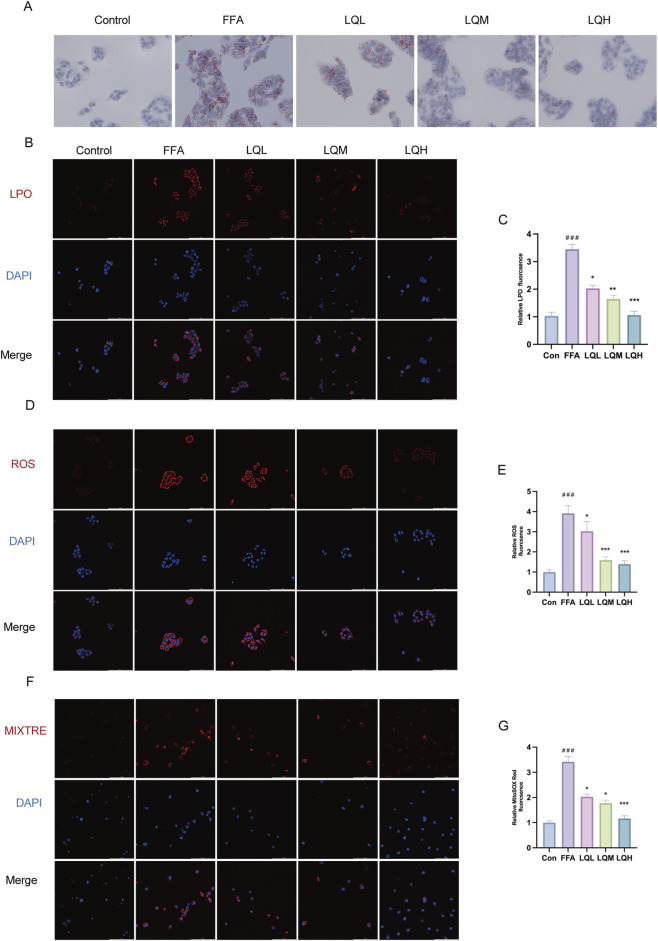
Oil Red O staining and injury-related fluorescence detection in HepG2 cells. **(A)** Oil Red O staining. **(B–G)** IF detection and statistical analysis of LPO, ROS and MIXTRE expression in HepG2 cells. Scale bar, 100 μm. All data are presented as mean ± SD from three independent experiments (n = 3 per group). ^###^ *P* < 0.001, compared to the Con group, ^*^ *P* < 0.05, ^**^ *P* < 0.01, ^***^ *P* < 0.001 compared to the FFA group.

To investigate the cytoprotective effects of LQ, we established a palmitic acid (FFA)-induced steatosis model in HepG2 cells. Oil Red O staining showed that LQ treatment reduced intracellular lipid content (Figure 7A). Results from specific fluorescent probes demonstrated that LQ significantly decreased the levels of total lipid peroxides (LPO) (Figures 7B,C) and reactive oxygen species (ROS) (Figures 7D,E). Detection using the Mito SOX™ Red probe (Figures 7F,G) revealed a marked decrease in mitochondrial superoxide fluorescence intensity following LQ treatment. These findings demonstrate that LQ not only reduces lipid accumulation effectively but also mitigates hepatocyte damage significantly.

### 3.7 LQ restores lipid metabolic balance by modulating AKT and FOXO1

**FIGURE 8:**
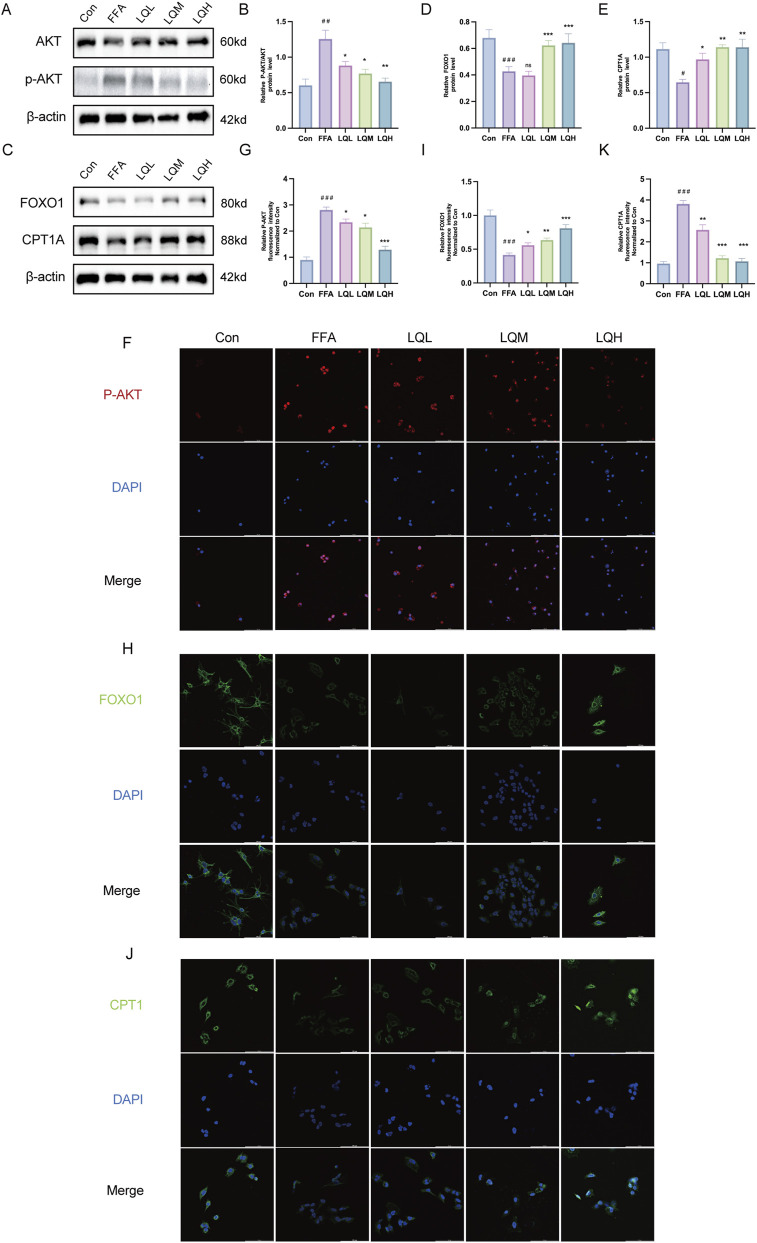
Western blot and immunofluorescence images of HepG2 cells. **(A)** Western blot analysis of AKT and p-AKT. **(B)** Quantification of the p-AKT/AKT ratio. **(C)** Western blot analysis of FOXO1 and CPT1. **(D,E)** Quantification of FOXO1 and CPT1A protein levels. **(F)** Immunofluorescence staining of p-AKT. **(G)** Quantification of p-AKT immunofluorescence intensity. **(H)** Immunofluorescence staining of FOXO1. **(I)** Quantification of FOXO1 immunofluorescence intensity. **(J)** Immunofluorescence staining of CPT1A. **(K)** Quantification of CPT1A immunofluorescence intensity. Scale bar, 100 μm. All data are presented as mean ± SD from three independent experiments (n = 3 per group). ^##^ *P* < 0.01, ^###^ *P* < 0.001, compared to the Con group, ^*^ *P* < 0.05, ^**^ *P* < 0.01, ^***^ *P* < 0.001compared to the FFA group.

**FIGURE 9:**
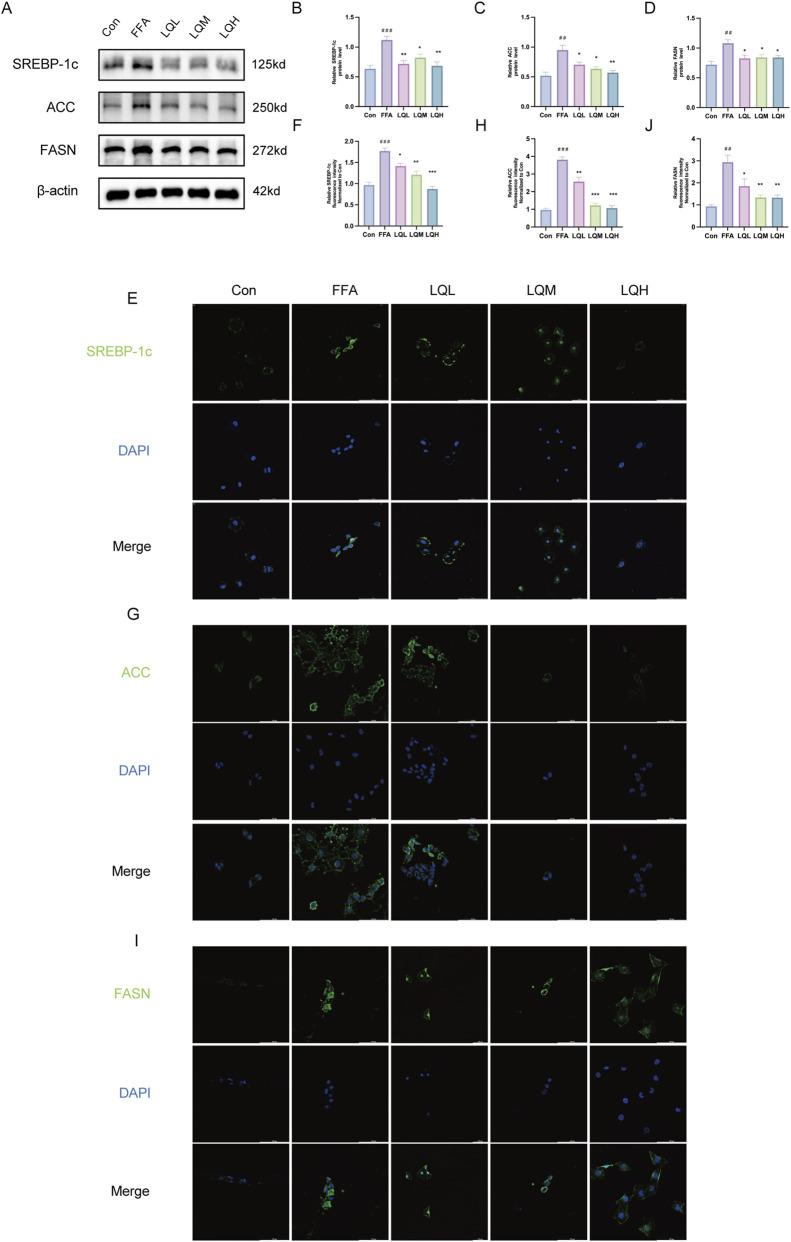
Western blot and immunofluorescence images of lipid metabolism proteins in HepG2 cells. **(A)** Western blot analysis of SREBP-1c, ACC, and FASN. **(B)** Quantification of SREBP-1c protein levels. **(C)** Quantification of ACC protein levels. **(D)** Quantification of FASN protein levels. **(E)** Immunofluorescence staining of SREBP-1c. **(F)** Quantification of SREBP-1c immunofluorescence intensity. **(G)** Immunofluorescence staining of ACC. **(H)** Quantification of ACC immunofluorescence intensity. **(I)** Immunofluorescence staining of FASN. **(J)** Quantification of FASN immunofluorescence intensity. Scale bar, 100 μm. All data are presented as mean ± SD from three independent experiments (n = 3 per group). ^##^ *P* < 0.01, ^###^ *P* < 0.001, compared to the Con group, ^*^ *P* < 0.05, ^**^ *P* < 0.01, ^***^ *P* < 0.001compared to the FFA group.

**FIGURE 10:**
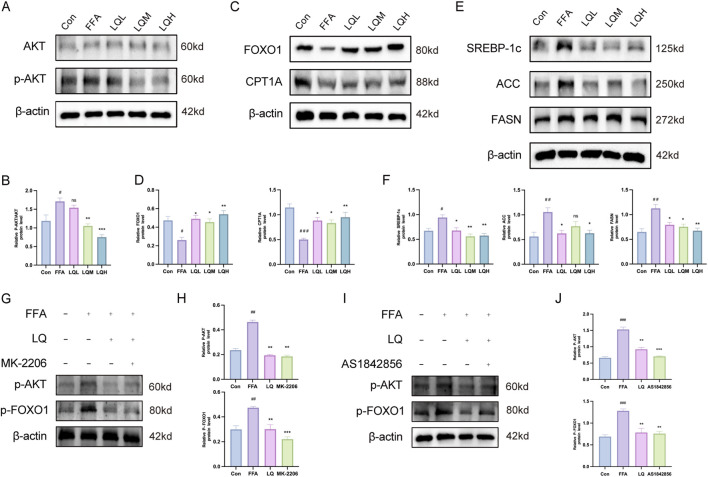
Western blot of HepG2 cells and AML12 cells. **(A)** Western blot analysis of AKT and p-AKT in AML12 cells. **(B)** Quantification of the p-AKT/AKT ratio in AML12 cells. **(C)** Western blot analysis of FOXO1 and CPT1 in AML12 cells. **(D)** Quantification of FOXO1 and CPT1A protein levels in AML12 cells. **(E)** Western blot analysis of SREBP-1c, ACC, and FASN in AML12 cells. **(F)** Quantification of SREBP-1c, ACC, and FASN protein levels in AML12 cells. **(G)** Western blot analysis of p-FOXO1 and p-AKT in HepG2 cells treated with MK-2206. **(H)** Quantification of the p-AKT and p-FOXO1 ratio in HepG2 cells treated with MK-2206. **(I)** Western blot analysis of p-FOXO1 and p-AKT in HepG2 cells treated with AS1842856. **(J)** Quantification of the p-AKT and p-FOXO1 ratio in HepG2 cells treated with AS1842856. All data are presented as mean ± SD from three independent experiments (n = 3 per group). ^##^ *P* < 0.01, ^###^ *P* < 0.001, compared to the Con group, ^*^ *P* < 0.05, ^**^ *P* < 0.01, ^***^ *P* < 0.001compared to the FFA group.

Western blot and immunofluorescence assays revealed that LQ treatment decreased the phosphorylation ratio of AKT (p-AKT/AKT) in HepG2 cells, indicating suppression of the AKT signaling pathway (Figures 8A,B,F,G). The expression of FOXO1 and CPT1A was lower in the non-alcoholic fatty liver disease model group but was significantly upregulated after LQ treatment (Figures 8C–E,I,K). Concurrently, LQ treatment markedly downregulated the expression of the key lipid synthesis regulator SREBP-1c and its downstream target proteins ACC and FASN (Figures 9A–D). These findings were further corroborated by immunofluorescence (Figures 9E–J). Western blot results from AML12 cells showed that LQ treatment reduced the phosphorylation ratio of AKT (p-AKT/AKT) in AML12 cells (Figures 10A,B), while the expression of FOXO1 and its downstream protein CPT1A was significantly upregulated following LQ intervention (Figures 10C,D). Compared with the control group, following LQ intervention, the expression of SREBP-1c, a key protein regulating lipid synthesis, was significantly reduced, and the protein expression levels of its downstream proteins ACC and FASN were decreased (Figures 10E,F). To further validate the mechanism of action of LQ, this study used AKT inhibitors and FOXO1 inhibitors in HepG2 cells. Western blot results showed that in the AKT inhibitor group following LQ intervention, the expression levels of p-AKT and p-FOXO1 were significantly downregulated, suggesting that LQ may regulate the phosphorylation-mediated inactivation of FOXO1 by influencing AKT phosphorylation (Figures 10G,H). In the FOXO1 inhibitor group following LQ intervention, the expression of p-FOXO1 was similarly affected by LQ, suggesting that LQ may exert a direct regulatory effect on FOXO1 (Figures 10I,J). Collectively, these data reveal a dual-action mechanism of LQ: it promotes CPT1A expression to accelerate fatty acid β-oxidation by regulating AKT phosphorylation and enhancing FOXO1 activity. Simultaneously, it suppresses SREBP-1c-mediated lipid synthesis pathways, thereby correcting lipid metabolism imbalance through bidirectional regulation.

### 3.8 LQ modulates mitophagy-associated markers in macrophages

**FIGURE 11:**
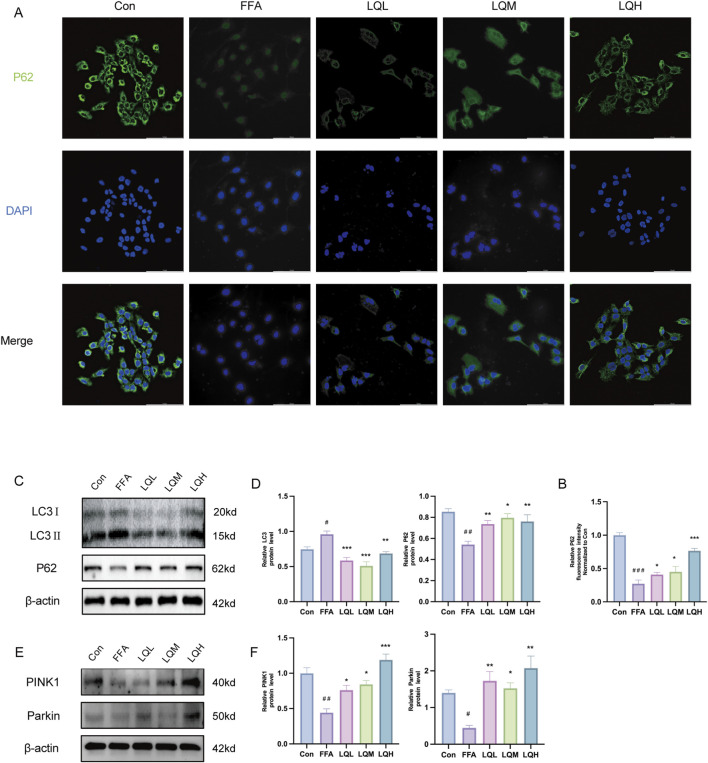
Western blot and fluorescence imaging of Raw264.7 cells **(A)** Immunofluorescence staining of P62. **(B)** Quantification of P62 immunofluorescence intensity. **(C)** Western blot analysis of P62 and LC3. **(D)** Quantification of P62 and LC3 protein levels. **(E)** Western blot analysis of PINK1 and Parkin. **(F)** Quantification of PINK1 and Parkin protein levels. Scale bar, 100 μm. All data are presented as mean ± SD from three independent experiments (n = 3 per group). ^#^ *P* < 0.05, ^##^ *P* < 0.01, ^###^ *P* < 0.001, compared to the Con group,^*^ *P* < 0.05, ^**^ *P* < 0.01, ^***^ *P* < 0.001 compared to the FFA group.

Single-cell analysis results indicate that hepatocytes exhibit robust signaling interactions with Kupffer cells and are associated with macrophage autophagy. In this study, using Raw264.7 macrophages, we investigated the effects of LQ on mitochondrial homeostasis in immune cells. Following palmitic acid (FFA) stimulation, cells exhibited changes in autophagy-associated protein levels: significant degradation of the autophagy substrate P62 protein and marked accumulation of LC3-II protein. LQ treatment effectively reversed these changes, restoring P62 protein levels to control group levels and reducing LC3-II accumulation (Figures 11A–D). Western blot results indicated that following LQ intervention, the expression levels of PINK1 and Parkin proteins were significantly increased compared to the model group (Figures 11E,F). These findings indicate that LQ modulates metabolic stress-induced excessive mitochondrial autophagy in immune cells. This action may indirectly promote overall liver microenvironment improvement by restoring mitochondrial functional homeostasis and enhancing the metabolic phenotype of hepatic immune cells.

## 4 Discussion

NAFLD is a chronic liver disorder closely associated with metabolic syndrome (He et al., 2024; Tsamos et al., 2024). Its global prevalence continues to rise, while effective targeted drug therapies remain limited (Abyadeh et al., 2022). The pathological core of this disease lies in disrupted hepatic lipid homeostasis, which involves dysfunction in multiple pathways, including fatty acid uptake, *de novo* synthesis, and oxidative catabolism (Sun et al., 2022; Zhang et al., 2024a; Yang et al., 2025). Consequently, developing drugs capable of multi-targeted intervention within this complex network is significant importance. This study focuses on LQ, one of the primary active components of the traditional Chinese medicinal herb licorice (Yuan et al., 2023; Kong et al., 2024; Xia et al., 2024). By integrating bioinformatics analysis, molecular docking simulations, and cellular and molecular experiments, we systematically investigated its role and mechanisms in improving NAFLD. Key findings indicate that LQ may exert protective effects by potentially influencing the AKT/FOXO1 signaling axis to regulate hepatic lipid metabolism while simultaneously influencing autophagy in hepatic macrophages.

The study began with an in-depth analysis of clinical samples from public databases to ensure the research direction’s relevance to the disease. Analysis of the transcriptome dataset GSE135251 identified the FOXO signaling pathway as a key pathway, whose activity was suppressed in NAFLD-specific pathological processes. In the NAFLD group, expression levels of the core transcription factor *FOXO1* within this pathway and its regulated fatty acid oxidation rate-limiting enzyme *CPT1A* (Tian et al., 2022; Hu et al., 2023; Sheppard et al., 2024) were significantly downregulated. Conversely, the key lipogenic transcription factor *SREBF1 *(Lee et al., 2024; Di Pasqua et al., 2026) and its directly regulated synthetic enzyme-encoding genes *FASN* and *ACACA* (Chu et al., 2021; Mu et al., 2021; Altuna-Coy et al., 2022) remained consistently upregulated. This clearly reveals the imbalance pattern in hepatic lipid metabolism during NAFLD: a diminished capacity for fatty acid oxidation clearance coupled with enhanced lipid synthesis and storage tendencies. As a key sensor and regulator of cellular energy and nutritional status, the downregulation of *FOXO1* suggests it may play a central role in NAFLD metabolic dysregulation. Based on the identified disease-associated characteristic genes, the study explored their upstream regulatory context by constructing a transcription factor regulatory network. Network analysis revealed that core metabolic genes such as *AKT1*, *FOXO1*, and *SREBF1* do not operate in isolation. Instead, they are embedded within a complex interactive network involving inflammatory factors like *STAT3* and *RELA* (Guo and Qu, 2021; Strum et al., 2021; Mangum et al., 2024; Tang et al., 2024), as well as stress and proliferation-related factors such as *MYC* and *TP53* (Dubois et al., 2022; Rodrigues et al., 2023). For instance, the lipogenic genes *SREBF1* and *FASN*, in addition to being regulated by their classical pathways, may also be directly regulated at their promoter regions by the oncogene *MYC*. *MYC* is frequently abnormally activated during the progression of NAFLD to more severe stages, which may partially explain the persistent hyperlipogenesis observed in the late stages of the disease. Furthermore, *AKT1* expression itself may be regulated by inflammatory mediators like *RELA*. This extensive network connectivity demonstrates, from a systems biology perspective, that lipid metabolism disorders in NAFLD are intricately intertwined with inflammatory signaling and cellular stress responses at the transcriptional level, forming a complex “metabolism-inflammation” interaction network. This also implies that intervening at any key node could yield systemic effects.

To gain a clearer understanding of the cellular origins and microenvironmental context of these changes, the study further integrated multiple single-cell transcriptomic datasets for analysis. Single-cell analysis identified major cell types in the liver, confirming hepatocytes as the primary site for abnormal lipid accumulation and metabolic gene alterations during NAFLD pathogenesis. A more detailed analysis of intercellular communication revealed significantly enhanced signaling interactions between hepatocytes and Kupffer cells under NAFLD conditions. This bioinformatic prediction functionally links the core metabolic cells (hepatocytes) with key effector cells (Kupffer cells), providing an explanation at the cellular level for metabolic dysregulation in NAFLD. Single-cell data further suggest that within hepatocytes, Netrin (Liao et al., 2022) promotes NAFLD progression by accelerating hepatic steatosis, potentially triggering fibrosis or even hepatocellular carcinoma, while simultaneously recruiting macrophages to exacerbate overall liver injury. Concurrently, PROS1(Yasmin et al., 2026) modulates lipid metabolism in hepatocytes, and together these factors influence NAFLD pathogenesis. In Kupffer cells, DHEAS (Moon et al., 2021; Zouhal et al., 2021; Wierman and Kiseljak-Vassiliades, 2022) and Netrin exhibit strong associations with mitochondrial autophagy. Macrophages undergo functional alterations, particularly showing signs of disrupted autophagy processes related to mitochondrial quality control, which provides direction for future research.

Subsequently, the study employed network pharmacology methods to identify potential drug targets capable of intervening in the aforementioned network. The analysis predicted that multiple targets of LQ overlap with NAFLD-associated genes, and AKT1 occupies a central hub position within the network. To investigate the potential direct binding between LQ and these targets from a physical structural perspective, molecular docking and molecular dynamics simulations were conducted. Computational results indicated that LQ may stably fits into specific binding pockets of AKT1 and FOXO1 proteins with favorable binding free energies. The LQ-protein complex appeared to maintained stable conformations throughout the simulation period, exhibiting persistent interactions such as hydrogen bonds. This computational evidence may provide theoretical support for LQ’s direct targeting of AKT1 and FOXO1 proteins and its potential to influence their active conformations or interactions.

This study conducted functional validation in cellular models based on bioinformatics analysis, molecular docking, and molecular dynamics simulation predictions. In a palmitic acid (FFA)-induced HepG2 hepatocyte steatosis model, LQ treatment demonstrated clear protective effects, significantly reducing intracellular lipid droplet accumulation, reducing oxidative stress marker accumulation within hepatocytes, and mitigating mitochondrial damage. At the molecular mechanism level, Western blot and immunofluorescence experiments confirmed that LQ reduces the phosphorylation level of AKT1 while increasing the total protein expression of FOXO1. This alteration not only led to upregulation of CPT1A—a key fatty acid oxidation enzyme activated by FOXO1—but also inhibited the SREBP-1c-mediated lipogenesis pathway, manifested by decreased protein levels of SREBP-1c and its downstream effectors ACC and FASN. The results of Western blot analysis of AML12 cells showed that LQ reduced the protein expression of p-AKT, increased the expression of FOXO1, and elevated the expression of CPT1A, a key protein in fatty acid β-oxidation, while simultaneously reducing the expression of SREBP-1c, a key protein in lipid synthesis, as well as its downstream targets ACC and FASN. To further validate the mechanism by which LQ regulates the AKT/FOXO1 signaling axis, we conducted functional intervention experiments using the AKT inhibitor MK-2206 and the FOXO1 inhibitor AS1842856 in free fatty acid (FFA)-stimulated HepG2 cells. Western blot results showed that in the AKT inhibitor group following LQ treatment, the expression levels of p-AKT and p-FOXO1 were significantly downregulated, suggesting that LQ may regulate FOXO1 inactivation by influencing AKT phosphorylation. In the FOXO1 inhibitor group following LQ intervention, the expression of p-FOXO1 was similarly affected by LQ, suggesting that LQ may exert a direct regulatory effect on FOXO1. These results support the hypothesis that LQ may activate AKT, thereby leading to FOXO1 inactivation and suppression of lipid synthesis gene expression. Furthermore, FOXO1 inhibition similarly attenuated the effects of LQ, indicating that FOXO1 is a key protein in the action of LQ. These inhibition experiments suggest that blocking AKT or FOXO1 affects the efficacy of LQ intervention, supporting the involvement of this pathway. These findings may delineate the action pathway of LQ in hepatocytes: by regulating the AKT/FOXO1 axis, it simultaneously enhances fatty acid β-oxidation and inhibits *de novo* lipid synthesis, thereby correcting lipid metabolism imbalance in a bidirectional manner. This mechanism aligns with the gene expression trends observed in clinical samples during transcriptomic analysis, indicating that LQ can reverse key metabolic defects associated with NAFLD at the cellular level.

Another line of inquiry in this study arose from the focus on Kupffer cells in single-cell analysis. Based on indications of potential autophagy dysregulation in Kupffer cells, targeted investigations were conducted in the RAW264.7 macrophage cell line. Experiments revealed that under metabolic stress simulated by palmitic acid (FFA), macrophages exhibited excessive activation of the mitochondrial autophagy pathway. This was characterized by accelerated degradation of the autophagy substrate P62 and accumulation of the autophagosome marker LC3-II. Concurrently, the expression of mitochondrial autophagy-related proteins PINK1 and Parkin was significantly downregulated, suggesting impaired mitochondrial autophagy function under lipotoxic conditions.LQ treatment effectively modulated this process, normalizing the levels of these autophagy markers. Mitochondrial autophagy is a critical process for maintaining cellular energy homeostasis and mitochondrial quality, and its dysregulation is closely associated with cellular metabolic dysfunction and inflammatory response. LQ treatment altered the protein levels of these autophagy-related markers, normalizing the expression of P62 and LC3-II and upregulating PINK1 and Parkin. This suggests that LQ may influence mitophagy-associated pathways under lipotoxic conditions, although direct assessment of autophagic flux is needed for confirmation. Within the NAFLD hepatic microenvironment, damaged hepatocytes communicate with Kupffer cells by releasing multiple signaling molecules. This induces a pro-inflammatory phenotype in these cells and thereby exacerbates tissue inflammation and injury. LQ’s ability to regulate macrophage autophagy status under metabolic stress suggests its effects may extend beyond hepatocytes themselves into the realm of hepatic immunoregulation. By stabilizing macrophage mitochondrial function and metabolic status, LQ may help suppress excessive inflammatory responses. This offers a new perspective on how LQ improves the overall hepatic microenvironment. Although direct hepatocyte-macrophage interactions were not validated in co-culture systems, this finding establishes a crucial experimental foundation for future investigations into how natural compounds can intervene in disease progression by regulating immune cell metabolism.

**FIGURE 12:**
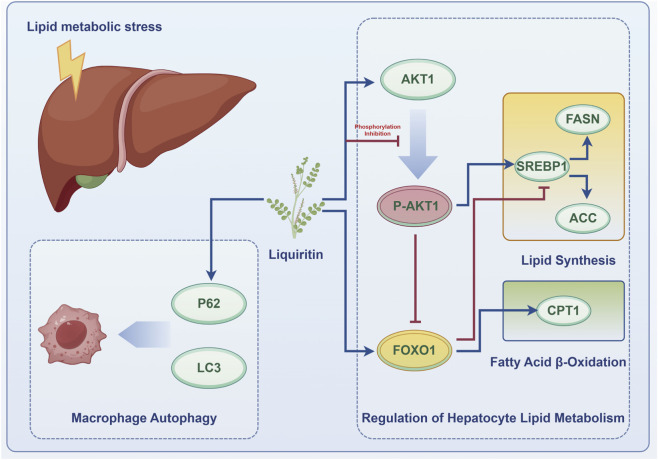
Mechanism diagram.

In summary, this study followed a research logic progressing from macro-to micro-level analyses and from observation to validation, progressively revealing the potential multidimensional mechanisms by which liquiritin counteracts NAFLD. First, clinical and single-cell transcriptomic analyses identified the core characteristics of FOXO1 signaling suppression and abnormal intercellular communication in the diseased state. Second, network pharmacology analysis, molecular docking, and docking simulations predicted and validated the AKT/FOXO1 axis as a potential direct target pathway for LQ. Subsequent cellular functional experiments confirmed that LQ restores hepatic lipid metabolism balance via this pathway. Finally, extending the mechanism exploration to Kupffer cells guided by single-cell data revealed LQ’s regulatory role in macrophage autophagy homeostasis. These interconnected findings collectively construct a comprehensive chain of evidence, suggesting LQ may not only directly correct hepatocyte metabolic defects but also indirectly improve the unfavorable tissue microenvironment by influencing the functional state of liver-resident immune cells. Naturally, this study is primarily limited to cellular-level mechanism exploration and represents a preclinical, exploratory investigation based on bioinformatics and *in vitro* experiments. The pharmacodynamic and pharmacokinetic characteristics of LQ in whole-animal models, along with direct evidence of its regulation of the hepatocyte-Kupffer cell interaction network, require future validation in NAFLD animal models and more complex co-culture systems. Furthermore, the precise effects of liquiritin on post-translational modifications of FOXO1 protein (e.g., acetylation) and other predicted key relationships within the regulated transcription factor network (e.g., *MYC* regulation of *FASN*) represent important scientific questions warranting further clarification. This study provides valuable preliminary evidence supporting LQ as a potential multi-target NAFLD therapeutic candidate acting on multiple cell types (Figure 12).

## 5 Conclusion

This study systematically investigates the molecular mechanisms by which LQ alleviates NAFLD through bioinformatics and cell biology approaches. Integrated analysis of whole-transcriptome and single-cell transcriptomic data reveals that FOXO pathway suppression and enhanced hepatocyte-macrophage interactions are key hallmarks of the disease. Network pharmacology and subsequent molecular dynamics simulations predicted AKT1 and FOXO1 as core targets, indicating that LQ may bind stably to both.

Functional validation in hepatocytes demonstrated that LQ reduces lipid accumulation by regulating the AKT/FOXO1 axis. It achieves this by inhibiting AKT phosphorylation and promoting FOXO1 expression, thereby downregulating lipid synthesis-related proteins SREBP-1c, ACC, and FASN while upregulating fatty acid oxidation protein CPT1A. Beyond hepatocyte metabolism, single-cell transcriptomic analysis suggests that LQ may also influence the expression of mitophagy-associated proteins in macrophages, potentially contributing to modulation of the immunometabolism microenvironment.

In summary, this study identifies the AKT/FOXO1 pathway as a potential key mediator of LQ’s actions, linking metabolic restoration with potential immune regulation and highlighting its potential as a multi-target therapeutic candidate for non-alcoholic fatty liver disease. Future research should focus on *in vivo* validation and refined mechanism exploration to advance its clinical translation.

## Data Availability

Transcriptome Analysis Datasets and Single-Cell Datasets derived from public domain resources, The data presented in this study are available in Gene Expression Omnibus (GEO) at https://www.ncbi.nlm.nih.gov/geo/, reference number GSE135251, GSE174748, GSE189175 and GSE212837. These data were derived from the following resources available in the public domain: https://www.ncbi.nlm.nih.gov/geo/query/acc.cgi?acc=GSE135251, https://www.ncbi.nlm.nih.gov/geo/query/acc.cgi?acc=GSE174748, https://www.ncbi.nlm.nih.gov/geo/query/acc.cgi?acc=GSE189175, https://www.ncbi.nlm.nih.gov/geo/query/acc.cgi?acc=GSE212837.
