# Supplementary material for: Liquiritin restores metabolic homeostasis in NAFLD by modulating AKT1/FOXO1 signaling
Source: Front Pharmacol. 2026 Jun 17;17:1809617. doi: 10.3389/fphar.2026.1809617 (PMC13318591; doi:10.3389/fphar.2026.1809617)

Supplementary Material

1 Supplementary Figure. 1 Transcriptomic data and network pharmacology.

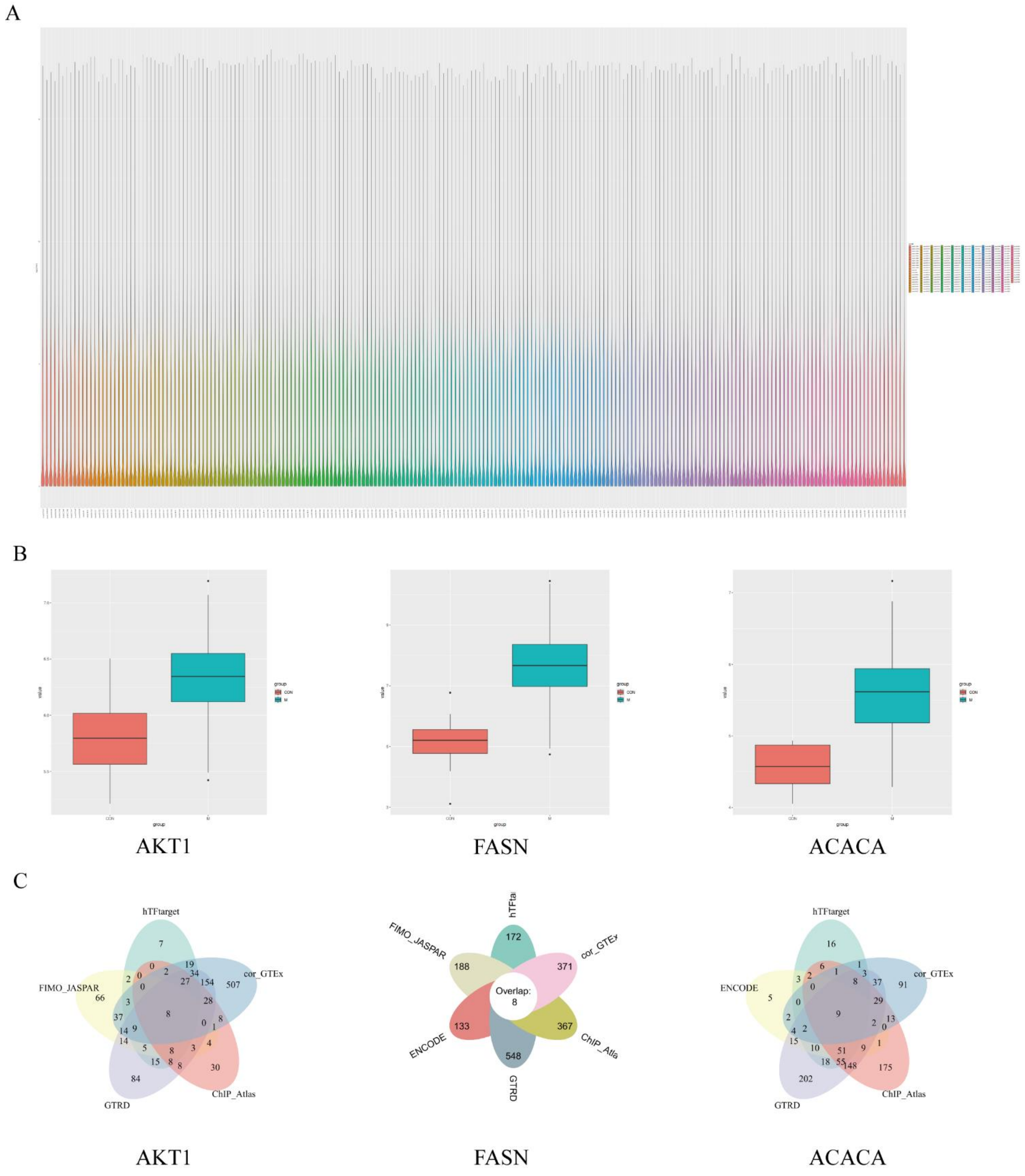

Supplementary Figure 1. (A) Violin plot analysis based on the GEO dataset GSE135251. (B) Gene expression analysis of *AKT1*, *FASN*, and *ACACA*. (C) Analysis of transcription factors regulating key genes: *AKT1*, *FASN*, and *ACACA*.

2 Supplementary Figure. 2 Cell Counting Kit-8 (CCK-8)

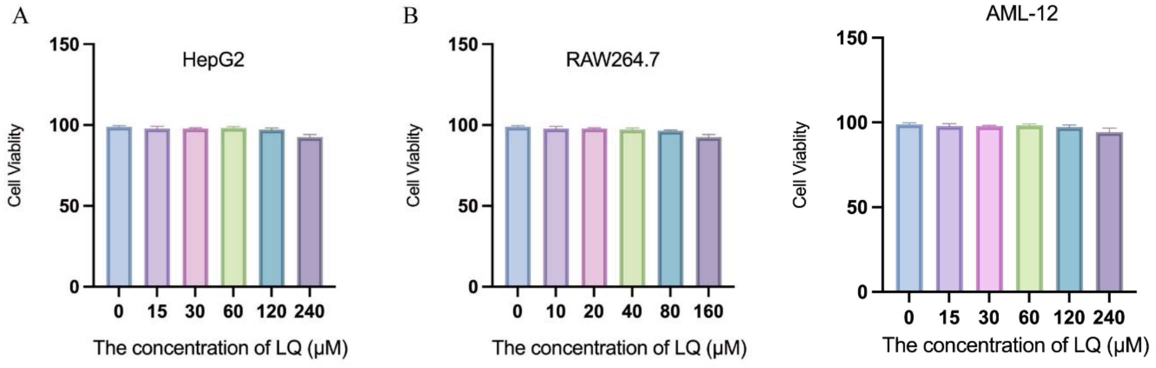

Supplementary Figure 2. (A) Cell Counting Kit-8 (CCK-8) of HepG2 cells. (B) CCK-8 of RAW264.7 cells. (C) CCK-8 of AML12 cells.

3 Supplementary Figure. 3 Single-Cell Gene Annotation Map

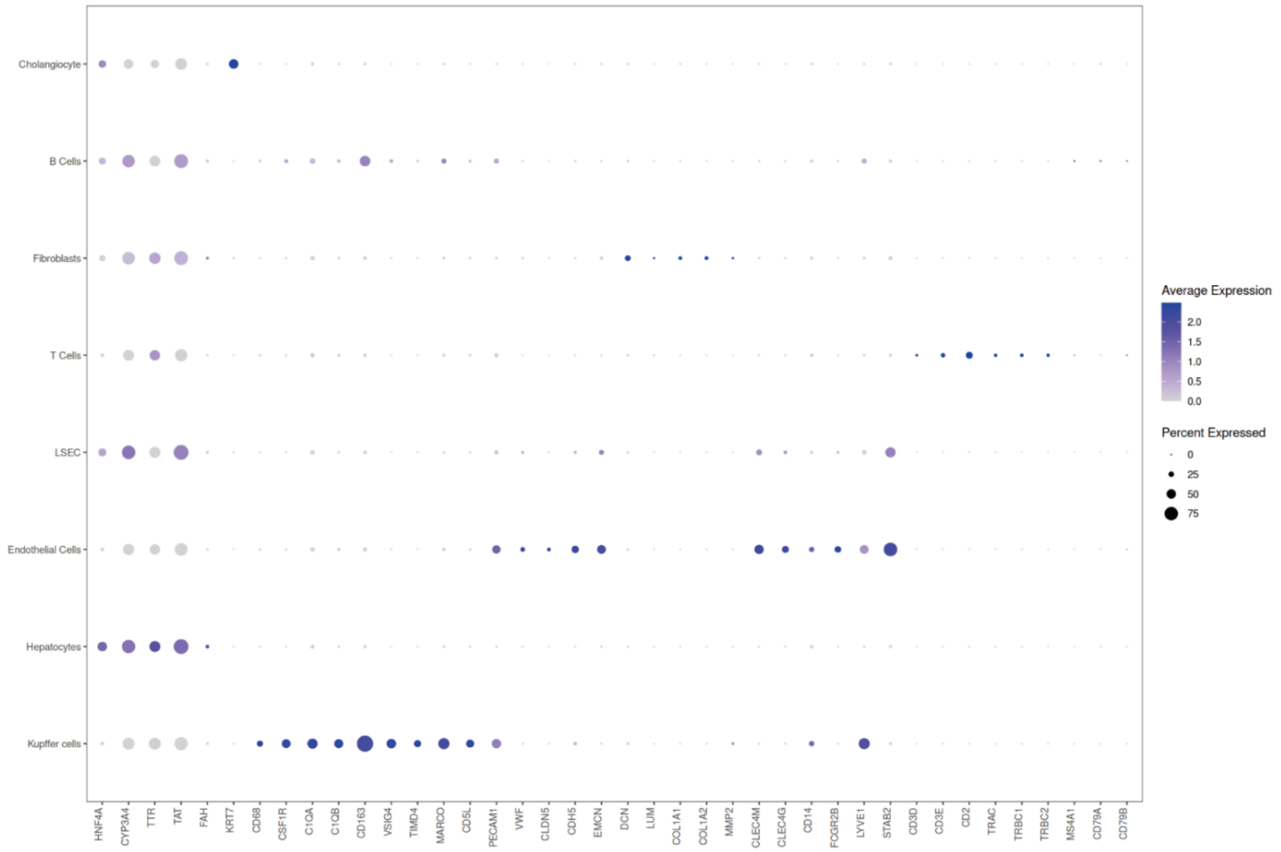

4 Supplementary Figure. 4 Cell-Ligand Interaction Map.

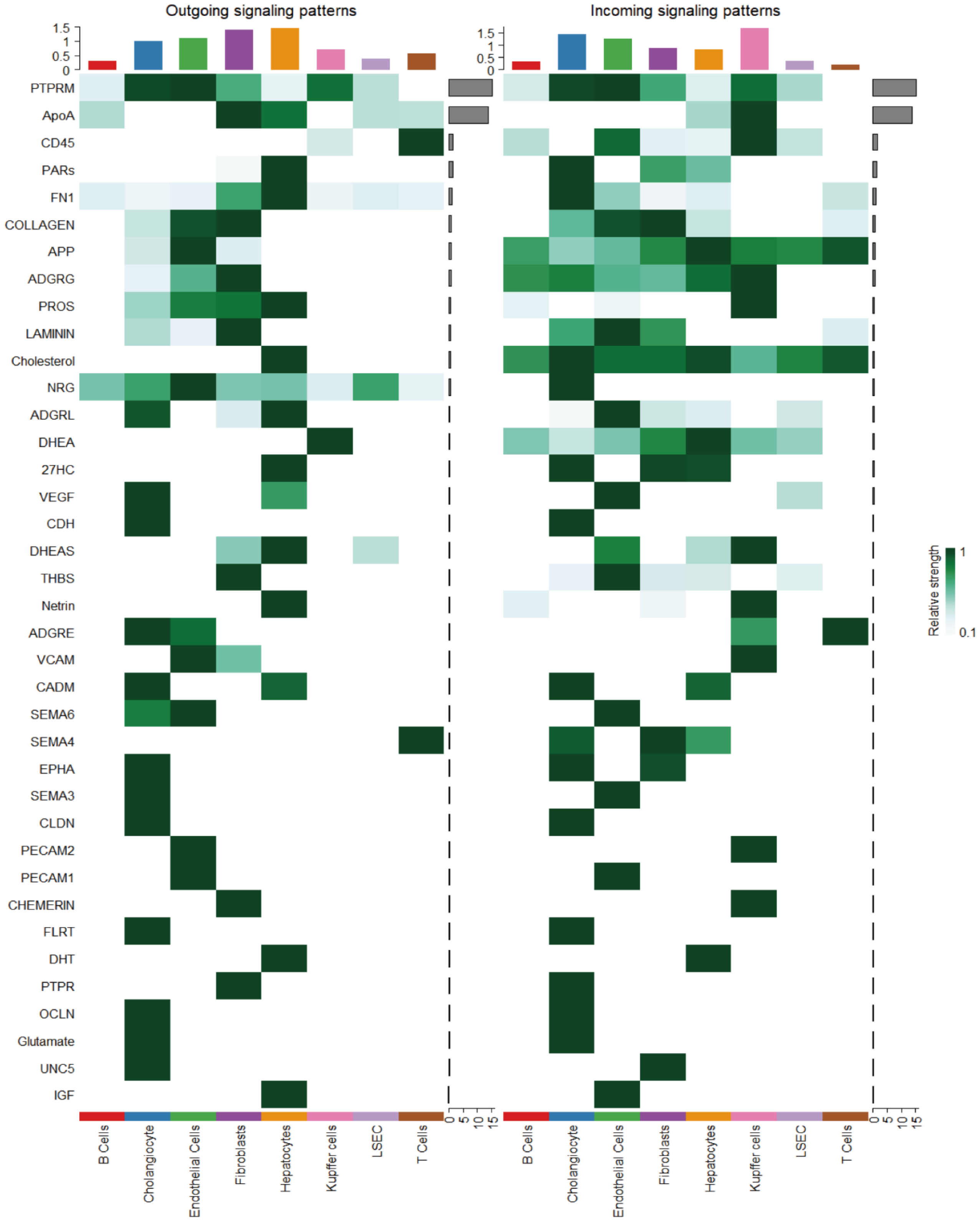

5 **Supplementary Figure. 5 DHEAS Signaling Pathway Interaction Diagram.**

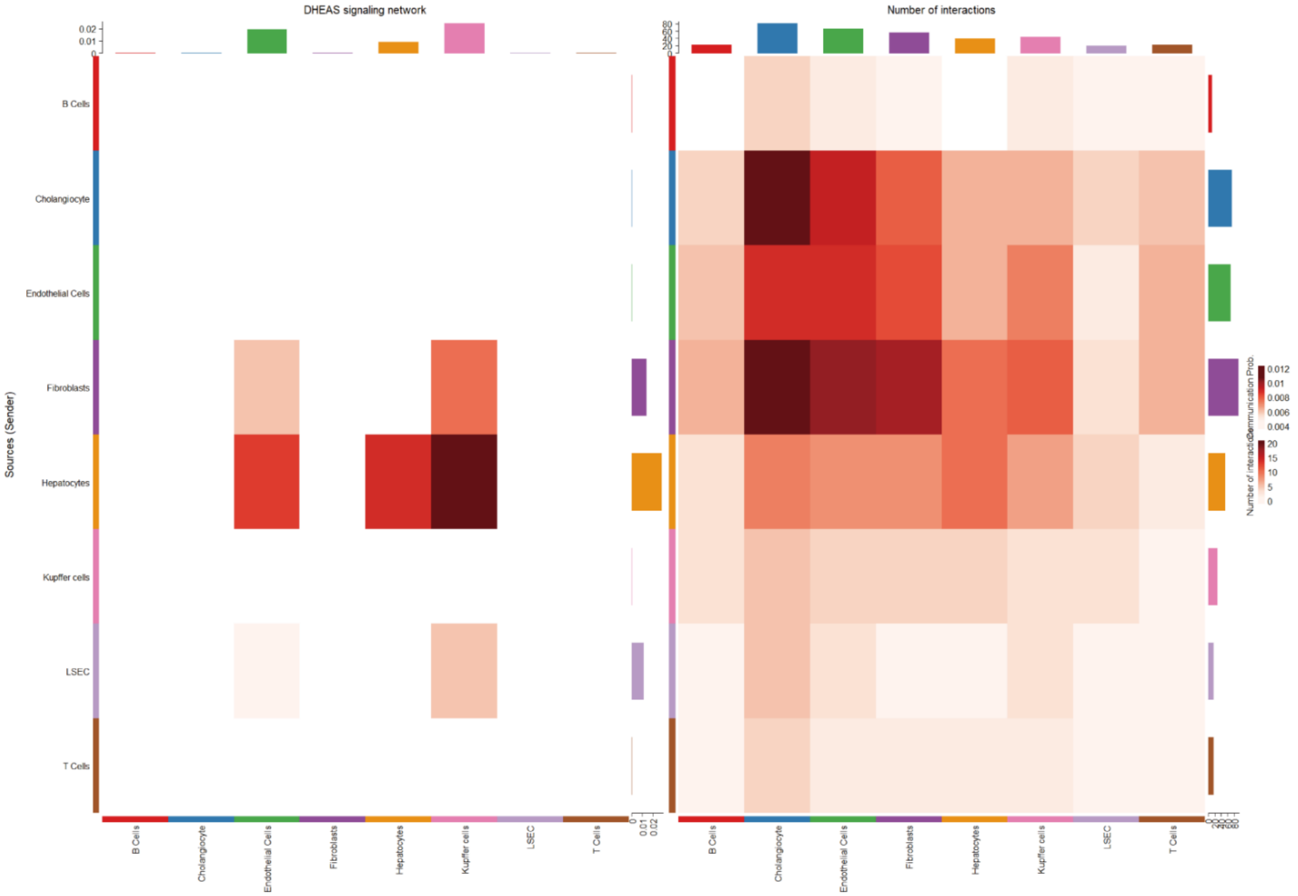

6 **Supplementary Figure. 6 Netrin Signaling Pathway Interaction Diagram.**

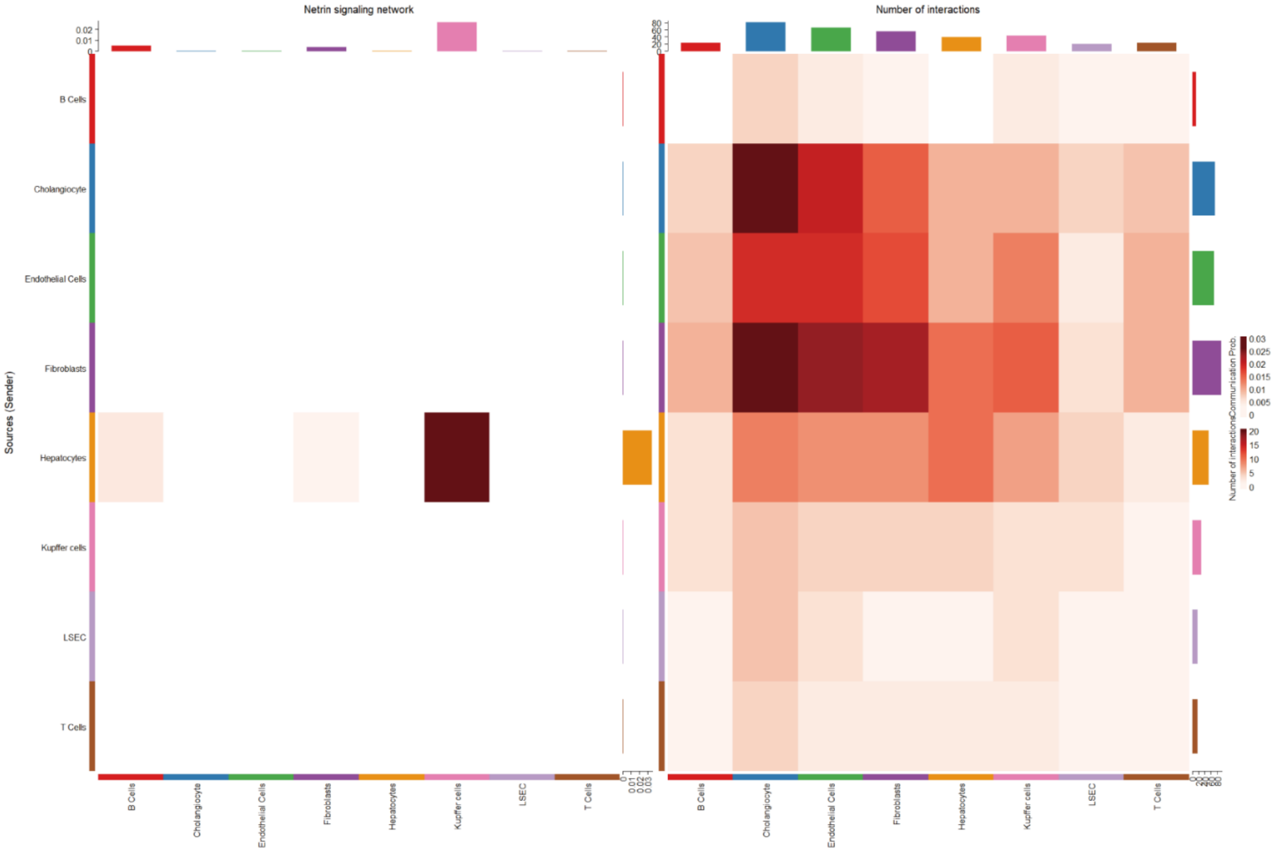

7 Supplementary Figure. 7 PROS Signaling Pathway Interaction Diagram.

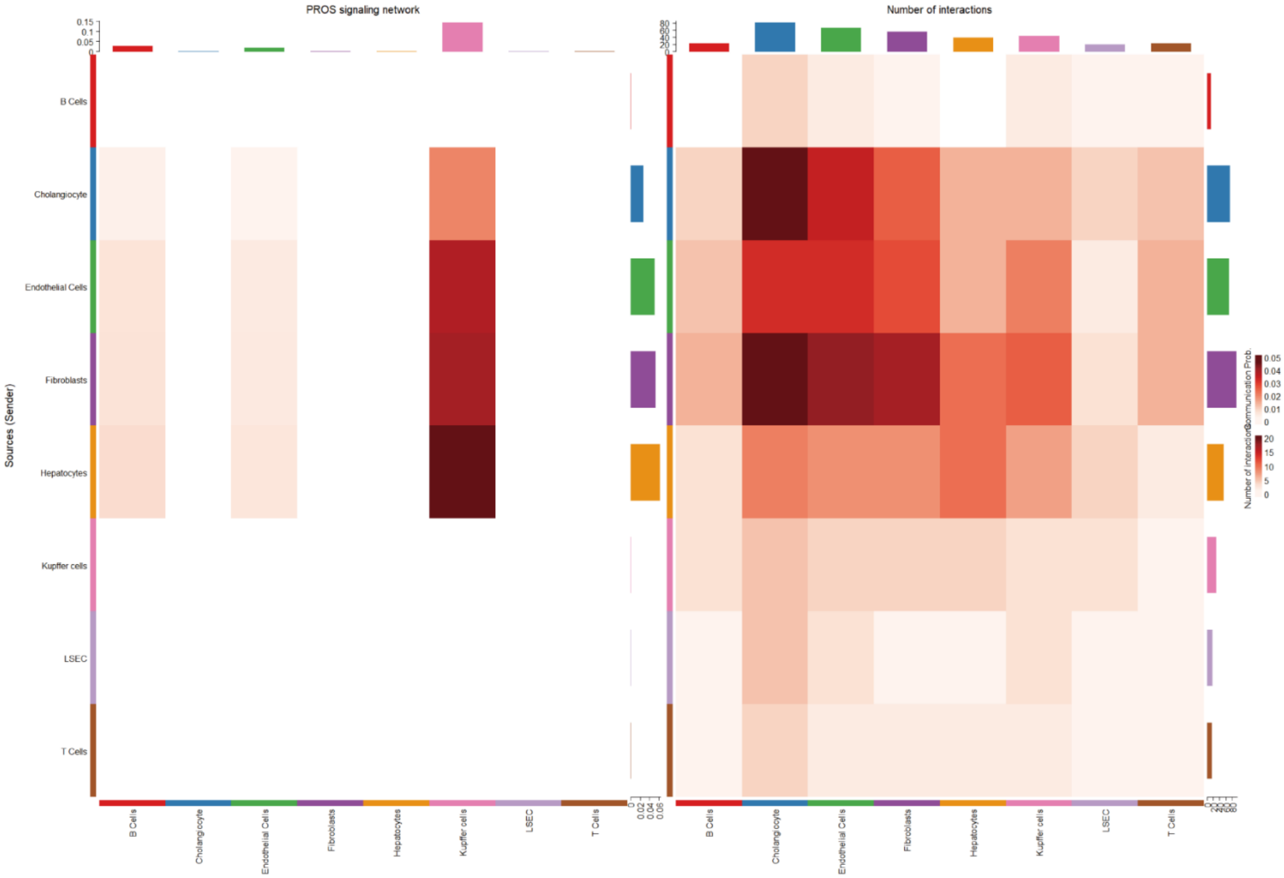

8 **Supplementary Figure. 8 CETSA Assay of Liquiritin and the FOXO1 Protein.**

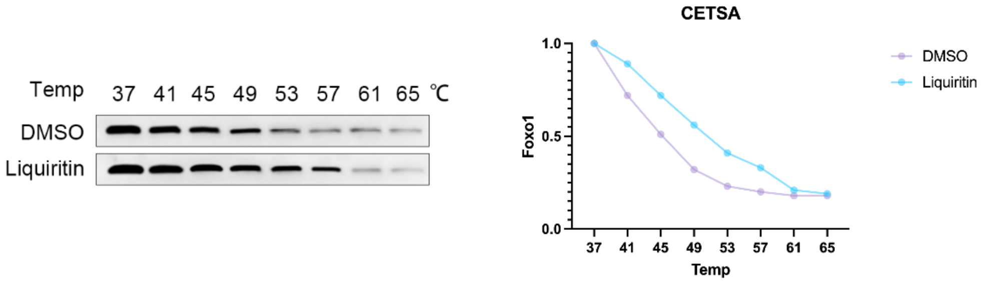

9 **Supplementary Figure. 9 Transcription Factor Table.**

Table X. Predicted transcription factor-target gene interactions involved in the regulation of key NAFLD-associated genes.

Transcription Factor	Target Gene	Predicted Binding Region	Data Source	Reference (PMID)
CTCF/ELF1/GABPA/SP1/KLF4/TFAP2A/NR2C2/RELA	AKT1	Promoter region	JASPAR / ENCODE	Wan et al., 2021; Wang et al., 2021
KLF11/RELA/REL/STAT3/ETV6/RORC/STAT1	FOXO1	Promoter region	JASPAR / hTFtarget	Ahmed et al., 2024; Albalawi and Khateeb, 2025; Li et al., 2025b
BCL6/STAT3/SP4/CEBPD/HNF4A/SREBF1	SREBF1	Promoter / Enhancer	JASPAR / ChIP-Atlas	Augustin et al., 2023
MYC/SP1/TCF12/E2F6/SP2/ZBTB7A/FOSL2/SREBF1	FASN	Promoter / Enhancer	JASPAR / ChIP-Atlas	Augustin et al., 2023
TP53/MEF2B/SP2/RORC/E2F8/MYC/TCF12/NFIC	CPT1A	Promoter region	JASPAR / ENCODE	Nishida et al., 2023; Sabe, 2023; Wang et al., 2024b
EP300/STAT1/TAF1/YY1/E2F4/RBBP5/SMC3/RELA/KDM4A	ACACA	Promoter region	JASPAR / hTFtarget	—

10 Supplementary Figure. 10 Additional Western blot bands.

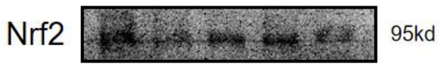

Supplement: Supplementary file 1 [file DataSheet1.pdf]
